# Spinel Aluminate‐Based Heterojunctions as Photocatalyst: A Review

**DOI:** 10.1002/open.202500473

**Published:** 2026-02-04

**Authors:** Ancy Kurian, Sumathi Shanmugam

**Affiliations:** ^1^ School of Advanced Sciences Department of Chemistry Vellore Institute of Technology Vellore Tamil Nadu India

**Keywords:** aluminates, dye degradation, metal oxides, photocatalysis, pollutant remediation

## Abstract

The growing demand for sustainable and efficient photocatalysts has driven extensive research into aluminate‐based materials due to their high stability, tunable electronic properties and promising photocatalytic performance. Nanocomposites derived from ZnAl_2_O_4_, MgAl_2_O_4_, CaAl_2_O_4_, and SrAl_2_O_4_ have shown remarkable potential in environmental remediation, particularly in the degradation of organic pollutants under light irradiation. This review explores the synthesis strategies, structural modifications, and performance enhancements of these aluminate‐based nanocomposites. Special emphasis is placed on the role of heterojunction engineering, ion doping, and band structure modulation in optimizing charge carrier dynamics and reducing recombination losses. Furthermore, the photocatalytic mechanisms of these materials are critically analyzed through the lens of energy band theory and interfacial charge transfer. The review also identifies current challenges and outlines future research directions, highlighting the potential of aluminate nanocomposites in advanced photocatalytic applications. By providing a systematic overview of their design principles and functional properties, this work aims to serve as a valuable resource for developing next‐generation photocatalysts with superior efficiency and stability.

## Graphical abstract

Spinel aluminates (MAl_2_O_4_; M = Zn, Mg, Ca, Sr) serve as robust platforms for designing heterojunction photocatalysts. Their stable lattices, tunable band structures, and efficient charge transport enable improved light‐driven carrier separation, significantly enhancing pollutant degradation efficiency in advanced photocatalytic systems.

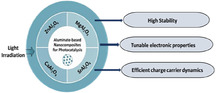

## 1 Introduction

The escalating global environmental crisis, particularly water pollution caused by organic contaminants, has emerged as one of the most pressing challenges of the 21st century. Industrial effluents, pharmaceutical wastes, and agricultural runoff continuously introduce recalcitrant organic pollutants into aquatic ecosystems, posing severe threats to both environmental sustainability and human health [1, 2, 3]. In response to this critical challenge, advanced oxidation processes (AOPs), especially photocatalytic degradation, have gained significant attention as environmentally benign and efficient treatment technologies [4].

Among various photocatalyt.ic materials, spinel aluminate nanocomposites (MAl_2_O_4_, where M represents divalent metal cations) have emerged as promising candidates due to their remarkable structural versatility, exceptional stability and tunable physicochemical properties. Key properties of spinel aluminates that contribute to their photocatalytic performance are charge carrier dynamics, high surface area, structural stability, and a tunable bandgap [5, 6]. These materials are characterized by their unique MB_2_O_4_ crystal structure, offer extensive possibilities for bandgap engineering, enhanced charge carrier separation, and improved photocatalytic efficiency through strategic compositional modifications and nanocomposite formation [7, 8, 9]. Recent advances in materials science and nanotechnology have revolutionized the development of spinel aluminate‐based nanocomposites, leading to unprecedented improvements in their photocatalytic performance. The incorporation of various functional components such as noble metals, semiconducting materials, carbonaceous materials, and other metal oxides has been shown to induce synergistic effects that help to address the inherent limitations of spinel aluminates such as ZnAl_2_O_4_, MgAl_2_O_4_, CaAl_2_O_4_, and SrAl_2_O_4_, particularly their wide bandgaps and rapid charge‐carrier recombination [10].

Despite significant advancements in photocatalysis, conventional semiconductor photocatalysts such as TiO_2_, ZnO and g‐C_3_N_4_ face inherent limitations, including wide bandgaps, inefficient visible‐light absorption, and rapid electron–hole recombination. These challenges have spurred interest in alternative materials, among which spinel aluminates (MAl_2_O_4_, M = Mg, Zn, Ca, Sr) have emerged as promising candidates due to their structural flexibility, high chemical stability, and tunable electronic properties. However, several critical research gaps persist. A major challenge is their limited visible‐light activity for spinel aluminates such as ZnAl_2_O_4_, MgAl_2_O_4_, CaAl_2_O_4_, and SrAl_2_O_4_; the optical bandgaps are very large (typically ranging from ~3.5  to >7 eV, depending on their structure and defect state), which restricts their efficient utilization under solar irradiation [11].

**FIGURE 1:**
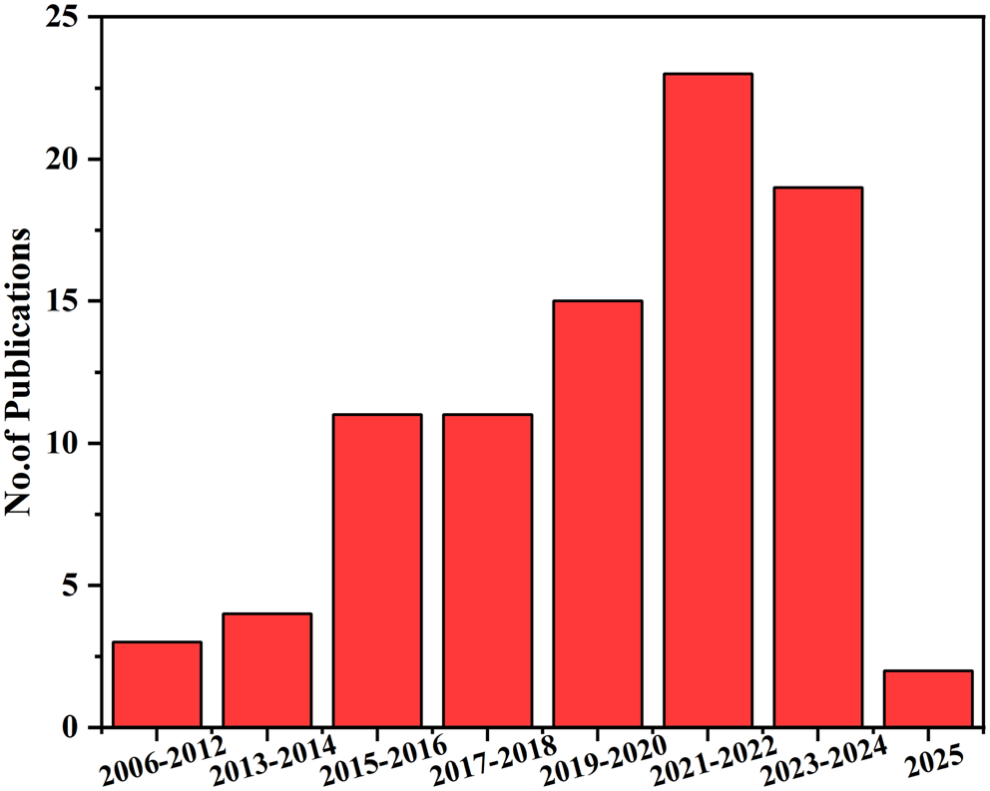
Publications on the photoactivity of different aluminates as photocatalyst.

Furthermore, while various studies have demonstrated the improved photocatalytic efficiency of aluminate‐based composites, the fundamental charge carrier dynamics, recombination pathways, and surface reaction mechanisms are still not well understood, necessitating in‐depth experimental and theoretical investigations. A comprehensive review that systematically covers the recent advancements in zinc aluminate, magnesium aluminate, calcium aluminate, and strontium aluminate‐based photocatalysts highlighting their synthesis strategies, reported heterojunctions, and nanocomposites formed via doping and interface engineering has been lacking. This review aims to fill that gap by critically discussing their photocatalytic mechanisms, evaluating the role of dopants and heterojunction formation, and offering a future perspective on overcoming existing limitations for enhanced photocatalytic performance. Figure 1 illustrates the number of research papers published on various aluminates used as photocatalysts. The data was gathered from search engines such as Web of Science, Scopus, and ScienceDirect on March 13, 2025, using individual keyword combinations including zinc aluminate as photocatalyst, magnesium aluminate as photocatalyst, calcium aluminate as photocatalyst, and strontium aluminate as photocatalyst. The resulting publication counts for each aluminate were then compiled and combined into a single, generalized plot to represent the overall publication trend.

## 2 Photocatalysis in Aluminates: Mechanisms, Heterojunction, and Functional Roles

Photocatalysis is a light‐induced catalytic process that utilizes semiconductors to degrade or transform chemical substances through oxidation and reduction reactions [12]. The foundational work in this field can be traced back to Fujishima and Honda, who in 1972 first demonstrated the photocatalytic splitting of water on a TiO_2_ electrode under ultraviolet light [13]. This groundbreaking discovery, often referred to as the Honda–Fujishima effect, marked the beginning of modern photocatalytic research and opened new pathways for environmental and energy applications [14].

**FIGURE 2:**
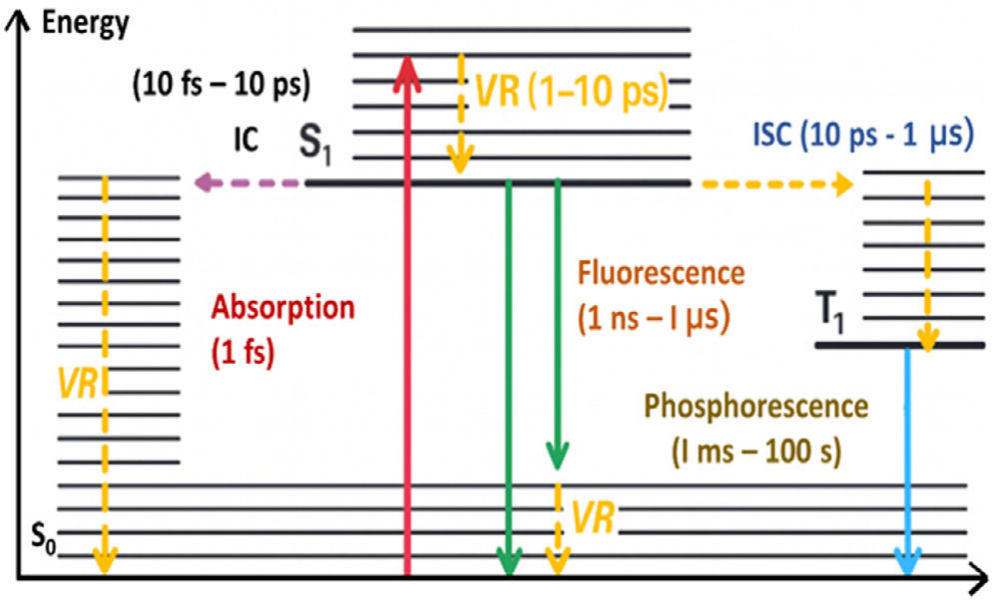
Energy transfer processes with different rates in a molecule after light activation. (S_0_ denotes the ground state singlet, S_1_ the first excited singlet state, and T_1_ the first excited triplet state).

A photocatalyst is typically a semiconductor material capable of absorbing photons to generate electron–hole pairs that drive surface redox reactions [15]. Its performance depends on factors such as bandgap energy, charge carrier dynamics, and surface properties [16]. Upon photon absorption, a molecule undergoes a transition from the ground state to the first singlet excited state within femtoseconds (fs). This photo‐induced excitation initiates a range of deactivation pathways, commonly represented through a simplified Jablonski diagram as shown in Figure 2. As described in the work by Zhang et al. [17], the excited charge carriers can follow several relaxation routes, depending on their energy and spin states:

a) Vibrational relaxation (VR): This is a fast nonradiative process (typically in the picosecond range), where excited electrons dissipate excess vibrational energy and settle into the lowest vibrational level of the excited electronic state. This energy is usually lost as heat.
b) Fluorescence: Once vibrationally relaxed, the molecule may return to its ground state via photon emission. This radiative transition, known as fluorescence, takes place over picoseconds to nanoseconds.
c) Internal conversion (IC): A nonradiative process where the excited molecule transitions from one electronic state to another of the same spin multiplicity (e.g., singlet‐to‐singlet). This typically occurs within femtoseconds to picoseconds.
d) Intersystem crossing (ISC): A transition between electronic states of different spin multiplicity, most commonly from a singlet to a triplet state. This process enables slower, spin‐forbidden deactivations like phosphorescence.
e) Phosphorescence: After ISC, the molecule in the triplet state may slowly return to the ground state by emitting a photon, resulting in phosphorescence. This can span from milliseconds to several seconds due to its forbidden nature.

Among various photocatalysts, spinel aluminates have attracted significant attention due to their exceptional chemical stability, tunable electronic properties, and wide bandgap characteristics [18]. The photocatalytic properties of spinel aluminates stem from their ability to generate electron–hole pairs upon photoexcitation, typically under UV or visible light irradiation. This process leads to the formation of reactive oxygen species at the material surface, which are crucial for photocatalytic applications [19]. The efficiency of these photocatalytic processes is influenced by various factors including particle size, morphology, surface area, porosity, bandgap, and surface modifications [20]. The ability to control these parameters through synthesis and modification techniques makes spinel aluminates highly versatile photocatalytic materials [21]. The modification capabilities of spinel aluminate provides numerous opportunities for property enhancement and optimization. These materials can be modified through various approaches including cation substitution, defect engineering, solid solution formation, and surface functionalization [22]. Additionally, their properties can be enhanced through doping with various elements, composite formation, morphology control, and size reduction to nanoscale dimensions [23, 24, 25, 26, 27, 28].

This flexibility in modification allows for the tailoring of properties to meet specific application requirements. These materials demonstrate excellent resistance to weathering, maintain stability in aqueous media, and are generally nontoxic in nature. Depending on the synthesis method employed, particle sizes can range from 20 nanometers to several micrometers, with morphologies varying from spherical to rod‐like [29], plate‐like [30], and rock like [31] structures. The surface area and porosity can be controlled through synthesis conditions, while the crystal phase purity, defect concentration, and agglomeration tendency can be optimized for specific applications [32, 33]. Their environmental friendliness and potential for recyclability make them attractive candidates for green chemistry applications and sustainable technologies.

To enhance their photocatalytic efficiency, heterojunction formation with other semiconductors is a strategic approach. Among the spinel aluminates, materials such as ZnAl_2_O_4_, MgAl_2_O_4_, CaAl_2_O_4_, and SrAl_2_O_4_ possess wide bandgaps, which limit their intrinsic visible‐light absorption. However, their conduction band (CB) and valence band (VB) positions can be modulated through heterojunction formation with narrow‐bandgap semiconductors. The key mechanisms governing charge separation and photocatalysis in these heterojunctions include type‐II, direct Z‐ scheme, and Schottky junction.

### 2.1 Type‐II Heterojunction Mechanism

**FIGURE 3:**
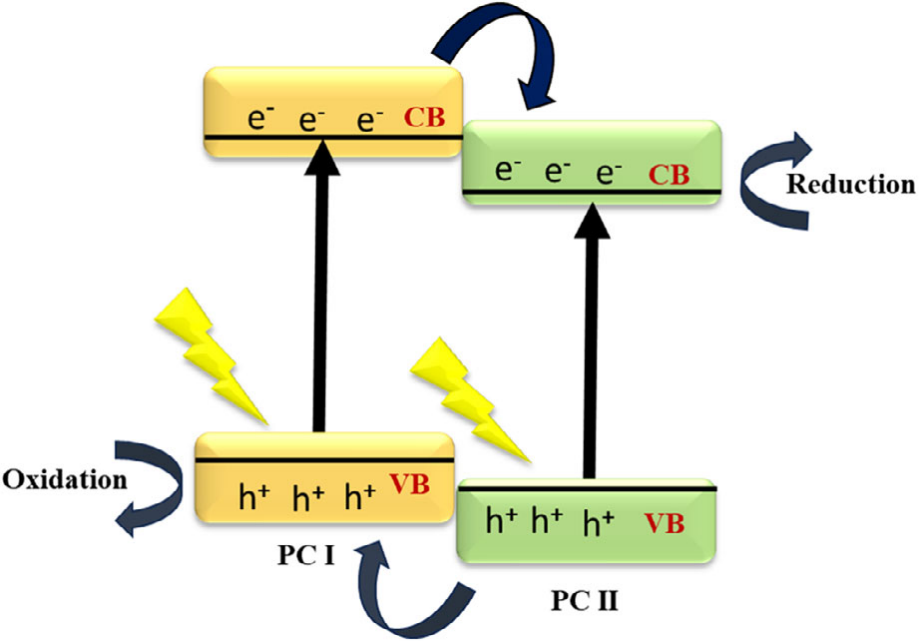
Type II mechanism.

In a type‐II heterojunction, the band edges of the two semiconductors are staggered in such a way that the CB of PC I semiconductor is positioned at a higher energy level than the CB of the PC II, while the VB follows the opposite trend. Upon illumination, photogenerated electrons transfer from the CB of the material with a higher CB energy to the CB of the material with a lower CB energy, while holes migrate in the opposite direction as illustrated in Figure 3 [34]. This spatial charge separation significantly reduces electron–hole recombination and enhances photocatalytic efficiency. For instance, in ZnAl_2_O_4_/BiPO_4_ heterojunctions, BiPO_4_ has a higher CB edge, allowing for efficient electron transfer from ZnAl_2_O_4_ to BiPO_4_, thereby facilitating enhanced redox reactions [35].

### 2.2 Direct Z‐Scheme Charge Transfer Mechanism

**FIGURE 4:**
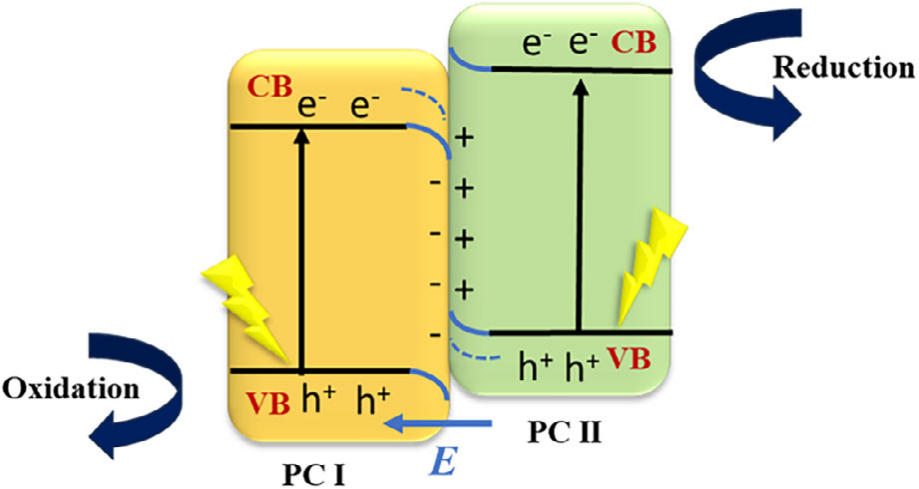
Direct Z‐scheme mechanism.

A direct Z‐scheme heterojunction mimics natural photosynthesis, where the charge carriers follow a stepwise migration, allowing for the retention of strong redox potential [36]. In this heterojunction system, electrons from the conduction band of PC I semiconductor recombine with holes in the valence band of PC II, enabling the selective retention of high‐energy charge carriers at CB of PC II and VB of PC I, thereby enhancing photocatalytic efficiency as illustrated in Figure 4 [37]. This mechanism is highly beneficial for pollutant degradation and hydrogen evolution reactions. For example, in a MgAl_2_O_4_/g‐C_3_N_4_ heterojunction, the photogenerated electrons from g‐C_3_N_4_ recombine with holes from MgAl_2_O_4_, while high‐energy electrons in MgAl_2_O_4_ and holes in g‐C_3_N_4_ remain available for redox reactions, improving the overall efficiency [38].

### 2.3 Schottky Junction and Surface Plasmon Resonance (SPR) Effects

**FIGURE 5:**
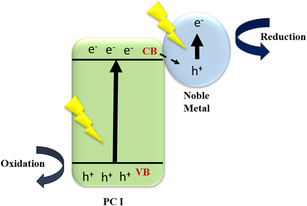
Schottky Junction.

When spinel aluminates are coupled with noble metals such as Ag or Au, Schottky junctions form at the interface, facilitating efficient charge separation. The noble metal serves as an electron sink, suppressing recombination and enhancing photocatalytic performance [39]. Additionally, the localized surface plasmon resonance (SPR) effect of noble metals enables strong visible‐light absorption, extending the spectral response of the heterojunction as shown in Figure 5. Incorporating rare‐earth metal dopants further enhances photocatalytic efficiency by introducing defect states, which act as charge trapping sites, prolonging charge carrier lifetimes. Rare‐earth elements can also induce up conversion photoluminescence, converting lower‐energy photons into higher‐energy ones, thereby improving visible‐light utilization [40]. These synergistic effects promote the generation of reactive oxygen species (ROS), such as hydroxyl radicals (^**•**^OH), superoxide radicals (^**•**^O_2_^−^), and singlet oxygen (^1^O_2_), facilitating pollutant degradation and microbial inactivation through improved charge separation and extended light absorption [41].

## 3 An Overview of Aluminates MAl_2_O_4_ [M = Mg, Zn, Ca and Sr]

Aluminates are a broad class of metal oxides consisting of aluminum and oxygen combined with various metal cations [42]. Their structural diversity, which includes spinel, monoclinic, and perovskite‐like phases, makes them highly versatile for applications in photocatalysis, luminescence, ceramics and energy storage [43, 44, 45]. The specific properties of aluminates, such as their thermal stability, mechanical strength, and electronic characteristics, are largely determined by the type and arrangement of metal cations within their crystal lattice [46, 47]. By modifying their composition, aluminates can be tailored for enhanced catalytic activity, optical performance, and structural resilience, making them valuable materials in both scientific research and industrial applications.

**FIGURE 6:**
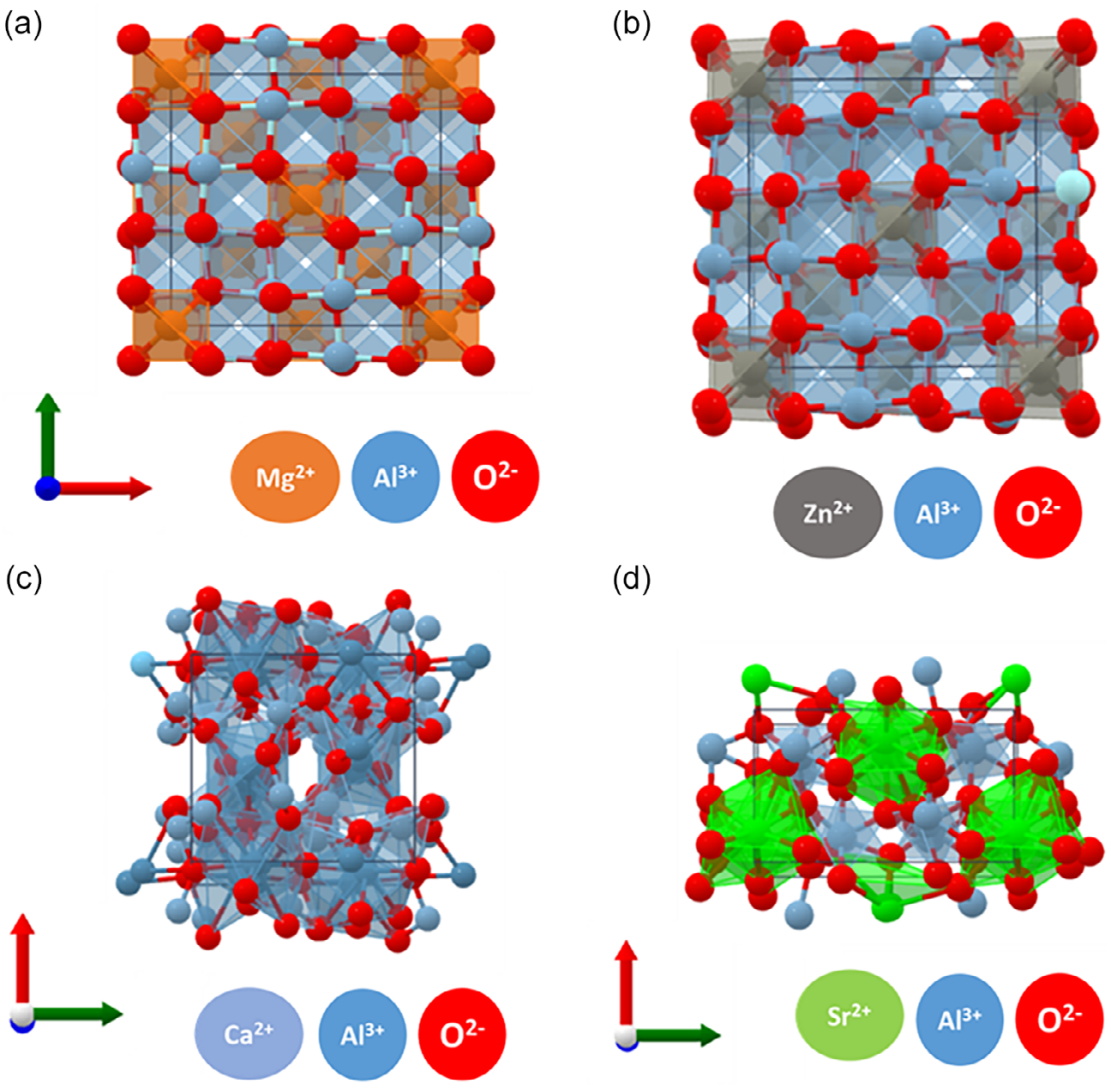
Cubic crystal structure of MgAl_2_O_4_ and ZnAl_2_O_4_ (a,b). Monoclinic crystal structure of CaAl_2_O_4_ and SrAl_2_O_4_ (c,d). Crystal structures were obtained from the open Materials Project database (MgAl_2_O_4_: MP‐ID ‐3536; ZnAl_2_O_4_: MP‐ID ‐2908; CaAl_2_O_4_: MP‐ID ‐14600; SrAl_2_O_4_: MP‐ID ‐3094).

Figure 6 illustrates the crystal structures of different aluminates, highlighting their unique cationic distributions within the aluminum‐oxygen framework. Magnesium aluminate (MgAl_2_O_4_) adopts a spinel structure, where Mg^2+^ ions occupy tetrahedral positions while Al^3+^ ions are arranged in octahedral coordination, contributing to its stability and catalytic efficiency as shown in Figure 6a [48]. Zinc aluminate (ZnAl_2_O_4_) shares a similar spinel arrangement, with Zn^2+^ ions substituting Mg^2+^ in tetrahedral sites, making it a promising material for photocatalysis and sensing applications (Figure 6b) [49].

Calcium aluminate (CaAl_2_O_4_) exhibits a monoclinic structure, where Ca^2+^ ions integrate into the Al‐O network, significantly influencing its hydration and reactivity, which are crucial for cementitious and refractory applications as depicted in Figure 6c [50]. Strontium aluminate (SrAl_2_O_4_) forms a distinct layered structure with Sr^2+^ ions embedded within the aluminum oxide framework, leading to remarkable luminescence properties used in long‐lasting phosphorescent materials as illustrated in Figure 6d [51]. The structural variations among these aluminates directly impact their physical, chemical, and functional properties, making them indispensable in various advanced material applications.

### 3.1 Magnesium Aluminate

Magnesium aluminate (MgAl_2_O_4_), also known as spinel, represents the archetypal member of the spinel group, crystallizing in a cubic structure with space group Fd3m [52]. The crystal structure features a face‐centered cubic arrangement of oxygen atoms, where Mg^2+^ ions occupy tetrahedral sites and Al^3+^ ions occupy octahedral positions [53]. This normal spinel structure exhibits a lattice parameter of *a* = 8.083 Å, characterized by perfect octahedral and tetrahedral coordination of the cations [54]. The material exhibits a wide bandgap of approximately 3.8 eV in its bulk form, which can be modified through various strategies [55]. The electronic structure features a valence band dominated by O 2p states and a conduction band primarily composed of Al 3s and 3p states, with Mg 3s states contributing at higher energies [56].

Conventional synthesis of MgAl_2_O_4_ via solid‐state reaction between MgO and Al_2_O_3_ typically requires elevated temperatures ranging from 800°C to 1600°C to achieve well‐crystallized products [57]. In contrast, solution‐based techniques such as sol–gel processing [58, 59] and coprecipitation [60, 61] offer the advantage of lower processing temperatures along with enhanced control over particle size and morphology. Recent advances in synthetic methodologies, including mechanochemical processing [62, 63, 64, 65] and plasma spray techniques [66, 67], have emerged as efficient alternatives, enabling rapid fabrication of materials with tailored structural and physicochemical properties.

To further enhance photocatalytic efficiency, surface engineering strategies like noble metal decoration and semiconductor heterojunction formation have proven effective by facilitating charge carrier separation and broadening the spectral absorption range [68, 69]. Additionally, precise morphological tuning through the formation of nanostructures such as nanoparticles, nanorods, or hollow spheres has been instrumental in increasing surface area and improving active site accessibility, thereby boosting catalytic performance. The incorporation of dopants, particularly transition metals and rare earth elements, has been shown to introduce intra‐band states, effectively narrowing the bandgap and improving the response under visible light irradiation [70, 71, 72]. Overall, the synthesis route plays a pivotal role in dictating critical material attributes such as surface area, crystallite size, and photocatalytic performance.

Ahmad et al. synthesized a MgAl_2_O_4_/MWCNT nanocomposite via chemical co‐precipitation for efficient dye degradation. Characterization confirmed its needle‐like morphology, reduced bandgap (3.02 to 2.78 eV), and enhanced electron mobility due to chemical defects. The nanocomposite demonstrated superior photocatalytic performance, degrading Methylene blue in 65 min, compared to 90 min for MgAl_2_O_4_. It also exhibited high stability and reusability, maintaining efficiency after six cycles [73].

#### 3.1.1 Heterojunctions with MgAl_2_O_4_

**FIGURE 7:**
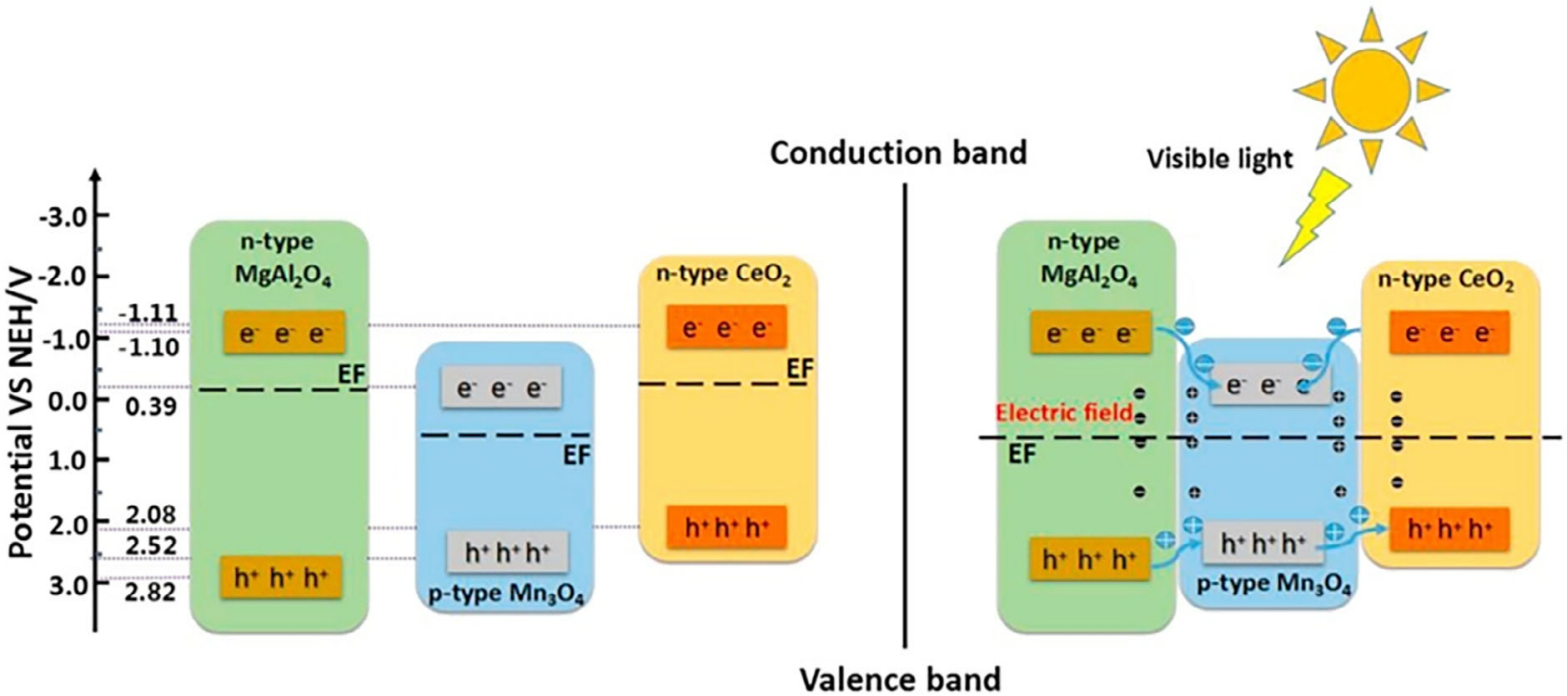
Photocatalytic mechanism of double p–n junction type MgAl_2_O_4_/CeO_2_/Mn_3_O_4_ heterojunction photocatalyst (Reproduced with permission [74]. Copyright 2021, Elsevier).

Recent advances include ternary composites such as MgAl_2_O_4_/CeO_2_/Mn_3_O_4_ have emerged as promising systems owing to their innovative double p–n junction architecture, which enables efficient charge carrier separation and migration as illustrated in Figure 7. The synergistic interaction between p‐type Mn_3_O_4_ and n‐type MgAl_2_O_4_ and CeO_2_ components facilitates spatial separation of redox sites, minimizing electron–hole recombination and accelerating degradation reactions. Furthermore, factors such as reduced particle agglomeration, ideal particle size distribution, and low optical absorption coefficient enhanced visible‐light harvesting and pollutant interaction. This study underscores the potential of multi‐junction heterostructure engineering as an effective strategy to surpass the limitations of conventional heterojunctions and develop highly efficient photocatalysts for environmental applications [74].

**FIGURE 8:**
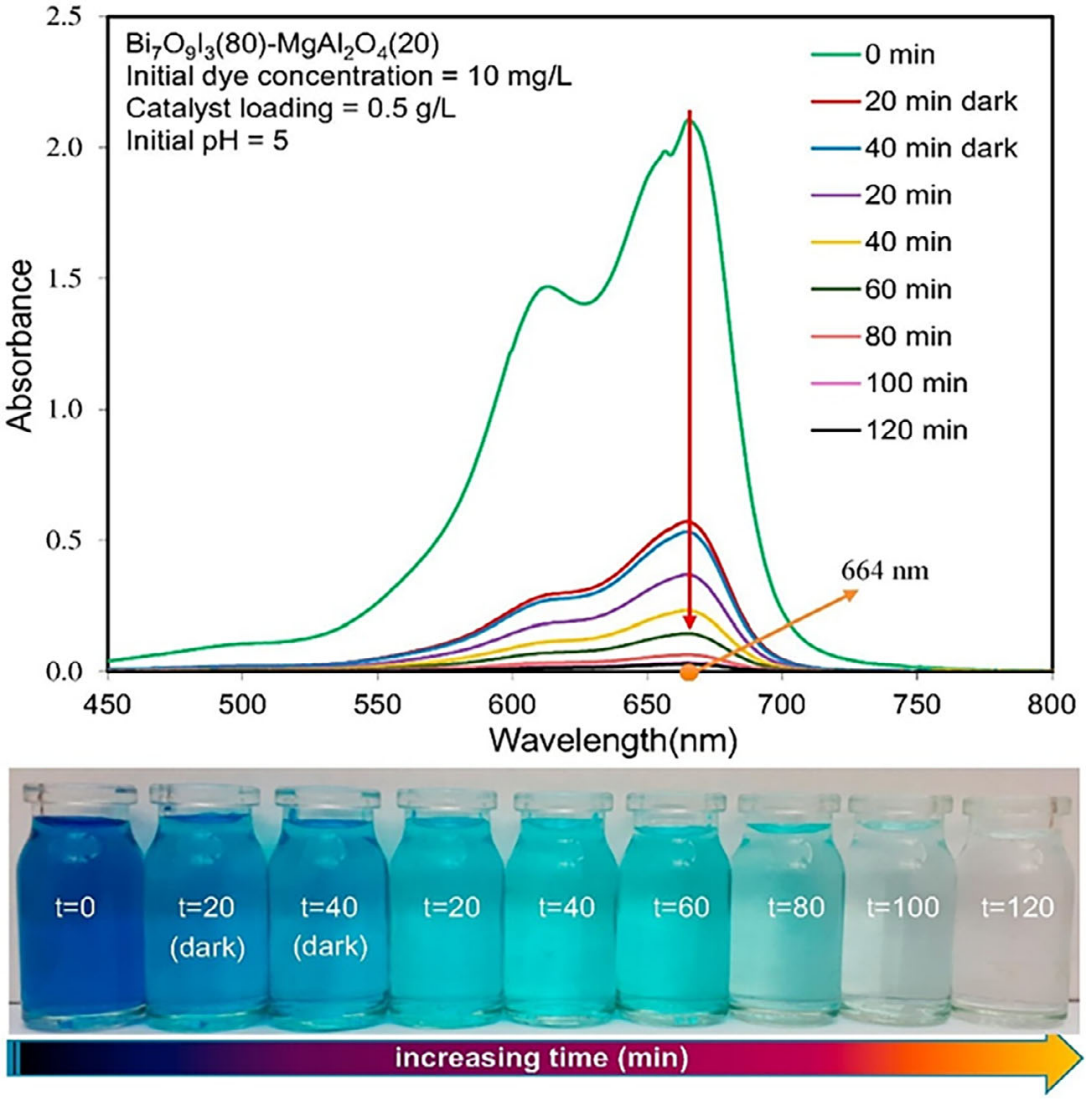
Photocatalytic activity of Bi_7_O_9_I_3_ (80)‐MgAl_2_O_4_ (20) for Methylene blue degradation (Reproduced with permission [75]. Copyright 2019, Elsevier).

The Bi_7_O_9_I_3_/MgAl_2_O_4_ photocatalyst (Figure 8) showed excellent performance in degrading Methylene blue, emphasizing the importance of adjusting the material composition. The study clearly shows that fine‐tuning the ratio between Bi_7_O_9_I_3_ and MgAl_2_O_4_ is key to improving photocatalytic activity. Among the tested ratios, the formation of a well‐matched p–n heterojunction between p‐type Bi_7_O_9_I_3_ and n‐type MgAl_2_O_4_ played a major role in enhancing charge separation and reducing recombination of electron–hole pairs, which directly contributed to better photocatalytic efficiency. Additionally, the nanocomposite benefited from the combined effects of its structural and surface properties, such as a well‐distributed pore network, high surface area, and uniform particle dispersion. These features improved light absorption and made active sites more accessible, allowing for more efficient interaction with dye molecules under visible light [75].

Salmasi et al., developed a Z‐scheme photocatalyst by coupling spinel MgAl_2_O_4_ nanospheres with thermally‐exfoliated g‐C_3_N_4_ nanosheets (TE‐GCN) using an isoelectric point‐assisted calcination method. The optimized conditions for the degradation of Reactive Red 195 (RR195) dye pH 3.0, 0.9 g/L catalyst dosage, and 70 min under simulated sunlight enabled nearly complete degradation. The enhanced photocatalytic activity was primarily due to the effective Z‐scheme charge transfer pathway, which promoted the spatial separation of photogenerated electrons and holes [38]. This is in contrast with the CeO_2_/MgAl_2_O_4_ composite, an effective n–n heterojunction between CeO_2_ and MgAl_2_O_4_ facilitates directional migration of charge carriers, thereby improving the spatial separation of oxidative and reductive sites. While CeO_2_ acts as the primary active component with strong redox capability, MgAl_2_O_4_ contributes to enhanced stability, increased adsorption capacity, and improved charge transport pathways due to its wide bandgap and excellent lattice compatibility [76]. Unlike the Z‐scheme mechanism, which preserves strong redox sites on both components, the n–n junction primarily enhances directional electron flow, leading to improved stability and adsorption but comparatively weaker oxidative capability_._

A similar trend is observed in the MgAl_2_O_4_/NiTiO_3_ nanocomposite synthesized via the sol–gel method forms a heterojunction that significantly enhances its photocatalytic performance. The heterojunction between the wide‐bandgap MgAl_2_O_4_ and the narrower‐bandgap NiTiO_3_ facilitates efficient separation and transfer of photogenerated electron–hole pairs, reducing recombination losses that typically limit photocatalytic efficiency in single‐phase materials [77]. Furthermore, WO_3_/MgAl_2_O_4_ nanocomposite photocatalyst was developed through a simple grinding technique involving MgAl_2_O_4_ spinel and WO_3_ powders. This physical mixing approach facilitated the intimate adhesion of WO_3_ particles onto the MgAl_2_O_4_ surface, promoting heterojunction formation. The formation of this heterojunction improved the generation and migration of reactive species such as hydroxyl radicals and photogenerated holes [78]. The degradation efficiencies of MgAl_2_O_4_‐based materials and their composites for various organic pollutants are summarized in Table 1.

**TABLE 1:** Photocatalytic performance of various magnesium aluminate‐based photocatalysts for pollutant degradation.

Photocatalyst	Pollutant	Light source	Irradiation time, min	Photocatalytic degradation, %	Reference
g‐C_3_N_4_/MgAl_2_O_4_	Methylene blue, Methyl orange	35 W Xe lamp	210, 300	100, 70	[79]
Mg_1−*x*_Zn_*x*_Al_2_O_4_	Methylene blue	Hg lamp	240	100	[80]
CeO_2_/MgAl_2_O_4_	Methylene blue	150 W Xe lamp	180	95.5	[76]
MgAl_2_O_4_	Methylene blue	350 W Xe lamp	100	99.5	[81]
MgAl_2_O_4_	Congo red	300 W Xe lamp	80	99.27	[82]
MgAl_2_O_4_	Methyl orange	400 W Hg lamp	140	87.1	[83]
Bi_7_O_9_I_3_/MgAl_2_O_4_	Methylene blue	400 W Halogen lamp	120	96.1	[75]
MgAl_2_O_4_/NiTiO_3_	Methyl orange	UV light	70	84	[77]
WO_3_/MgAl_2_O_4_	Methylene blue	18 W UV light	300	86	[78]
MgAl_2_O_4_/CeO_2_/Mn_3_O_4_	Methylene blue	150 W Xe lamp	180	94.6	[74]

### 3.2 Zinc Aluminate

Zinc aluminate (ZnAl_2_O_4_), commonly known as gahnite, represents a classical spinel structure with space group Fd3m [84]. The crystal structure consists of a cubic close‐packed arrangement of oxygen ions, where Zn^2+^ ions occupy tetrahedral sites and Al^3+^ ions occupy octahedral positions in the lattice [85]. This normal spinel structure exhibits a lattice parameter of *a* = 8.086 Å, with the unit cell containing 32 oxygen atoms, 16 Al^3+^ ions in octahedral sites, and 8 Zn^2+^ ions in tetrahedral sites [86].

The synthesis of ZnAl_2_O_4_ can be achieved through various methodologies, each offering specific advantages in controlling the final product characteristics. Traditional solid‐state reaction between ZnO and Al_2_O_3_ requires high temperatures (1000°C–1200°C) but offers simplicity and scalability [87]. Sol‐gel methods provide excellent control over particle size and morphology, typically operating at lower temperatures (600°C–900°C) and resulting in higher surface area products [88]. ZnAl_2_O_4_ synthesized via the sol–gel method at a calcination temperature of 500°C exhibited a crystallite size of 12 nm, a high surface area of 78.59 m^2^/g, and a bandgap of 3.49 eV. When tested for photocatalytic degradation of 15 mg/L Methylene blue (MB), it achieved a 98.9% degradation efficiency with a degradation rate constant of 0.0236 min^−1^, completing the degradation within 80 min [89].

The microemulsion‐derived ZnAl_2_O_4_, calcined at 850°C, exhibited a crystallite size of 21.7 nm, a moderate surface area of 42.9 m^2^/g, and a bandgap of 4.15 eV. This catalyst demonstrated excellent photocatalytic activity, achieving 100% degradation of 20 mg/L Methylene blue degradation [90]. Hydrothermal synthesis offers superior control over crystal growth and morphology. Other methods such as solution combustion synthesis, co‐precipitation, and microwave‐assisted techniques have emerged as efficient and rapid synthesis with controlled particle characteristics [91, 92, 93, 94].

Green synthesis has emerged as a sustainable alternative for producing nanomaterials with minimal environmental impact. Vinitha et al. [95] reported the synthesis of zinc aluminate nanoparticles via a microwave‐assisted green route, utilizing *Opuntia* dillenii plant extract as a natural reductant and stabilizer. This method effectively reduced synthesis time while enhancing material purity and crystallinity. The material exhibits a wide bandgap of ≈3.8–4.9 eV, which can be strategically modified through various approaches [96]. The valence band consists predominantly of O 2p and Zn 3d states, while the conduction band is primarily composed of Al 3s and 3p states [97].

Notable advances include the development of visible‐light–active systems through bandgap engineering, improved charge separation through heterojunction formation, and enhanced surface properties through novel synthesis approaches. However, challenges persist, including the need for better visible light utilization and improved quantum efficiency. A silver‐modified ZnAl_2_O_4_ composite (5 wt% Ag/ZnAl_2_O_4_) synthesized via the microemulsion method and calcined at 850°C demonstrated a crystallite size of 17.7 nm and a bandgap of 4.06 eV. The photocatalyst exhibited outstanding performance, achieving 100% degradation of 20 mg/L Methylene blue within 100 min [98].

#### 3.2.1 Heterojunctions With ZnAl_2_O_4_

**FIGURE 9:**
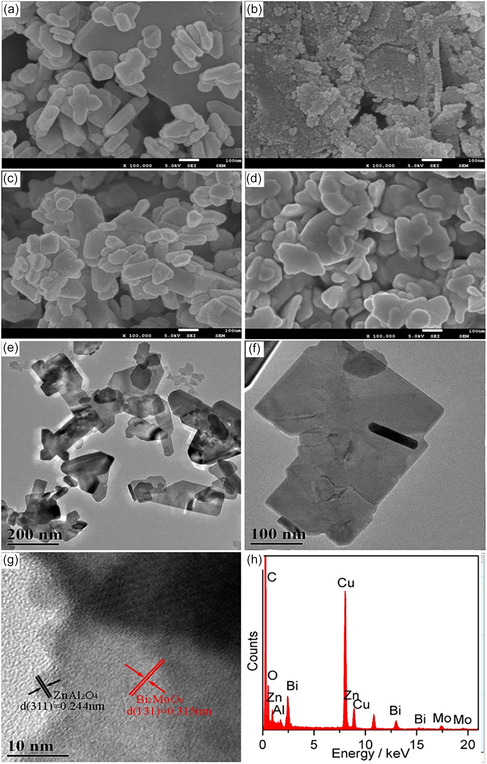
SEM images of pure Bi_2_MoO6, ZnAl_2_O_4_, 0.5 wt% ZnAl_2_O_4_/Bi_2_MoO_6_, 5 wt% ZnAl_2_O_4_/Bi_2_MoO_6_, low‐magnification TEM image of 5 wt% ZnAl_2_O_4_/Bi_2_MoO_6_, HRTEM of 5 wt% ZnAl_2_O_4_/Bi_2_MoO_6_, EDS of 5 wt% ZnAl_2_O_4_/Bi_2_MoO_6_ (Reproduced with permission [99]. Copyright 2020, Elsevier).

Semiconductor heterojunctions such as ZnAl_2_O_4_/Bi_2_MoO_6_ (Figure 9) heterostructure was developed through co‐precipitation and hydrothermal techniques, enabling effective photodegradation of Methylene blue under UV irradiation. The photocatalytic performance was significantly influenced by the ZnAl_2_O_4_ content, 0.5 wt% ZnAl_2_O_4_/Bi_2_MoO_6_ composite showing the highest degradation efficiency an 86.36% removal rate, outperforming both individual components. This enhanced activity was attributed to the efficient charge separation facilitated by the heterojunction structure, which reduced recombination of photogenerated electron–hole pairs [99]. Tian et al. reported ZnAl_2_O_4_/BiPO_4_ nanocomposites exhibited markedly improved photocatalytic performance under UV light, primarily attributed to the efficient separation and transfer of photoinduced charge carriers across the heterojunction interface. The integration of ZnAl_2_O_4_ into the BiPO_4_ matrix facilitated effective charge separation due to the interfacial interaction between the two semiconductors. The optimized composite composition and heterostructure contributed to enhanced charge carrier dynamics and structural stability. Additionally, the material showed potential applicability in the treatment of real industrial wastewater, and its performance was further influenced by the presence of H_2_O_2_, indicating its suitability for advanced environmental remediation strategies [35].

Dhinakaran et al. developed a ZnAl_2_O_4_/CeO_2_ photocatalyst via a facile hydrothermal method, tailored for the effective degradation of organic pollutants. The study emphasized the crucial role of the compositional ratio between ZnAl_2_O_4_ and CeO_2_ in determining the photocatalytic efficiency. This enhanced photocatalytic activity was mainly attributed to the reduced bandgap energy of the composite (2.6 eV), which was lower than that of the individual components [100]. In line with the trend observed across ZnAl_2_O_4_ ‐based heterojunctions where the coupled semiconductor largely dictates the charge–transfer mechanism, Ghribi et al. reported the successful synthesis of a ZnO‐ZnAl_2_O_4_ heterojunction photocatalyst derived from a layered double hydroxide precursor for the solar‐light‐driven degradation of metronidazole (MNZ), an emerging pharmaceutical contaminant. The composite structure, consisting of ZnO and ZnAl_2_O_4_ nanoparticles, formed a type‐II heterojunction. The intimate contact between ZnO and ZnAl_2_O_4_ nanoparticles facilitates efficient electron–hole separation, thereby enhancing photocatalytic performance under neutral pH conditions. Additionally, the presence of abundant surface hydroxyl groups provides a hydrophilic interface that improves pollutant adsorption and accelerates the degradation process. This distinct combination of type‐II charge alignment and surface–driven interaction further broadens the mechanistic diversity of ZnAl_2_O_4_‐based photocatalysts [101].

In a recent study, ZnAl_2_O_4_/Bi_2_O_3_ heterojunctions were fabricated via a simple grinding approach, demonstrating high efficiency for pollutant degradation. The enhanced performance was attributed to the formation of a direct Z‐scheme heterostructure, which facilitated spatial charge separation by retaining high redox potentials of both photogenerated electrons and holes. This configuration proved superior to conventional type‐II systems by reducing charge recombination and promoting the generation of reactive oxygen species essential for pollutant breakdown. The integration of ZnAl_2_O_4_ with Bi_2_O_3_ effectively harnessed both UV and visible light, underscoring its potential for advanced photocatalytic applications [102]. The photocatalytic efficiency of different zinc aluminate‐based photocatalysts for pollutant degradation is detailed in Table 2, highlighting their performance under various experimental condition.

**TABLE 2:** Photocatalytic performance of various Zinc aluminate‐based photocatalysts for pollutant degradation.

Photocatalyst	Pollutant	Light source	Irradiation time, min	Photocatalytic degradation, %	Reference
ZnAl_2_O_4_/BiPO_4_	Methylene blue	100 W Hg lamp	180	86	[35]
ZnAl_2_O_4_	Methylene blue	sunlight	90	98	[89]
ZnAl_2_O_4_	Indigo carmine	UV visible	60	99	[103]
PtO @ ZnAl_2_O_4_	Hg (II)	300 W Xenon	60	100	[104]
ZnALDH	Methyl orange	(40 W/230 V Philips	120		[105]
Lychee biochar‐ ZnAl_2_O_4_	Ibuprofen	UV lamp, Hg 125 W	60 ads + 60 photo	100	[106]
ZnAl_2_O_4_/ZnO	Methyl orange	UV lamp	60	95.5	[107]
g‐C_3_N_4_/ ZnAl_2_O_4_	Methyl orange	500 W Xe lamp	120	96	[108]
ZnAl_2_O_4_/Bi_2_MoO_6_	Methylene blue	100 W Hg lamp	180	86	[99]
ZnO/ZnAl_2_O_4_	Metronidazole	Sunlight	300	95	[101]
Polyaniline‐ ZnAl_2_O_4_	Crystal violet	18 W UV lamp	120	96.58	[109]
ZnAl_2_O_4_/CeO_2_	Methylene blue Methyl orange Rhodamine B	250 W UV lamp	180	98 98 99	[100]
ZnAl_2_O_4_/Bi_2_O_3_	Rhodamine B Tetracycline	250 W UV; 500 W visible	90 (RhB),60 (TC); 180	99 (RhB), 98 (TC); 95 (RhB), 92 (TC)	[102]

### 3.3 Calcium Aluminate

Calcium aluminate (CaAl_2_O_4_) represents a significant member of the spinel‐like structure family, exhibiting a distinctive monoclinic crystal system. The structure is characterized by a complex three‐dimensional network of corner‐sharing AlO_4_ tetrahedra and AlO_6_ octahedra, with Ca^2+^ ions occupying the interstitial sites [110]. This unique arrangement results in specific unit cell parameters: *a* = 8.700 Å, *b* = 8.092 Å, *c* = 15.191 Å, and *β* = 90.17°, which contribute to its distinctive properties and applications in various fields [111].

Sol‐gel methods, operating at relatively higher temperatures (1000°C–1500°C), provide better control over particle size and morphology [112]. Solution combustion synthesis offers superior control over crystal growth [113]. Calcium magnesium aluminate (Ca_2_Mg_2_Al_28_O_46_) nanoparticles were synthesized using aloe vera gel as a bio‐template in a green fuel‐assisted solution combustion method, where metal nitrates were incorporated with the plant extract and subjected to thermal treatment at 550°C followed by calcination at 950°C [114]. The material exhibits a density of 2.98 g/cm^3^ and a high melting point of 1600°C, with surface areas ranging from 15–45 m^2^/g depending on the synthesis method [115]. Its electronic structure features a bulk bandgap of 5.6–6.0 eV, which can be strategically reduced to 3.4–3.8 eV in nanostructured forms [116]. This bandgap modification is crucial for enhancing its photocatalytic activity and expanding its potential applications.

**FIGURE 10:**
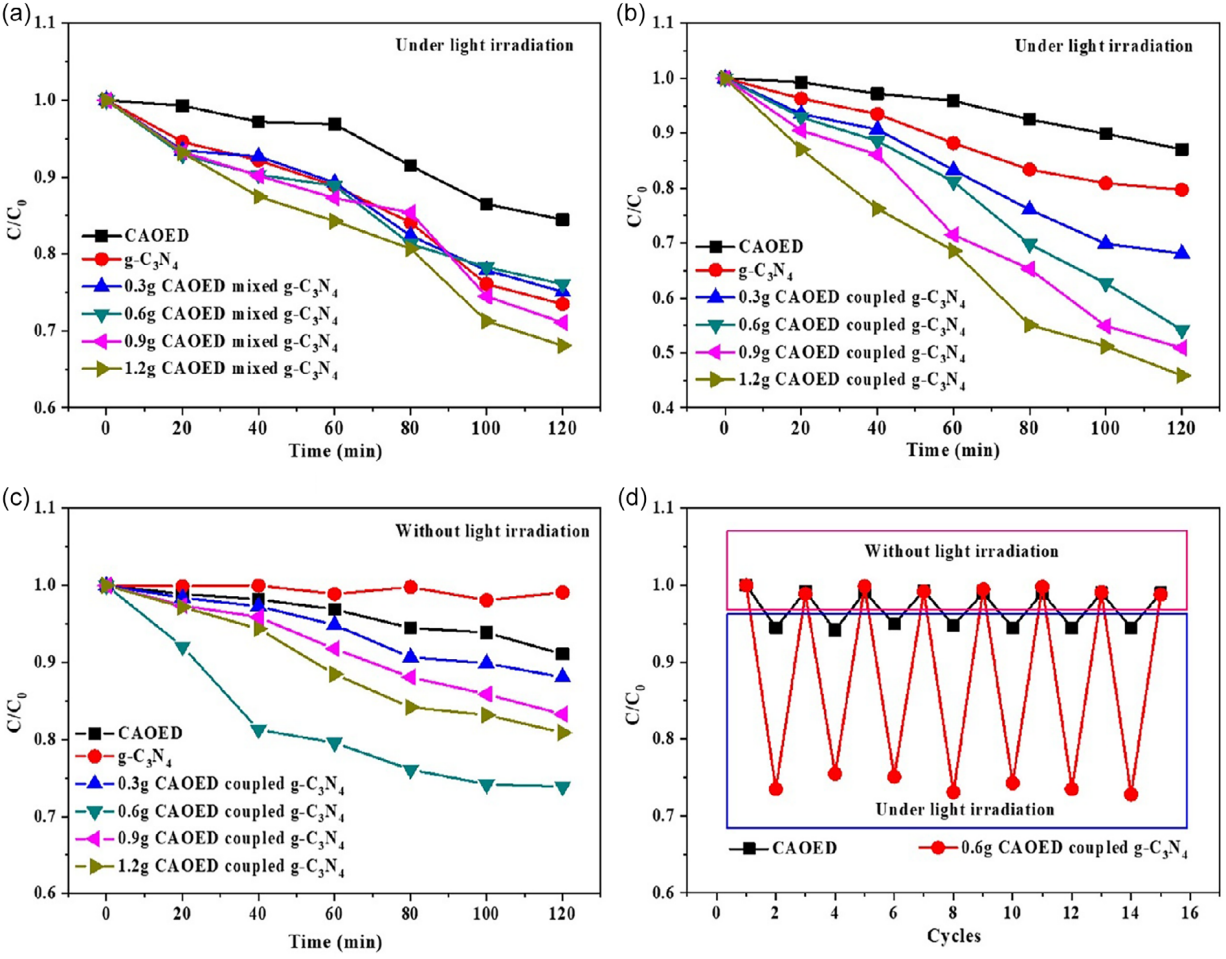
Photocatalytic degradation of the CAOED, g‐C_3_N_4_, and CAOED‐mixed g‐C_3_N_4_ QDs composites on the MO under light on, photocatalytic degradation of the CAOED, g‐C_3_N_4_, and CAOED‐coupled g‐C_3_N_4_ QDs composites on the MO under light on and off, and photocatalytic degradation of the MO dye using CAOED and CAOED‐coupled g‐C_3_N_4_ QDs composites under repeated light on and off (Reproduced with permission [117]. Copyright 2020, Elsevier).

Zhou et al. reported a round‐the‐clock photocatalyst by coupling CaAl_2_O_4_: Eu^2+^, Nd^3+^ (CAOED) long‐lasting phosphor with g‐C_3_N_4_ quantum dots (QDs) for continuous Methyl orange (MO) degradation even in darkness as shown in Figure 10. The CAOED‐coupled composite maintained photocatalytic activity for over 3 h after light removal, attributed to persistent luminescence from CAOED. The stored energy could be recharged and recovered, ensuring sustained performance. This irradiation‐free photocatalysis was driven by crystal lattice defects in CAOED, which enhanced visible‐light utilization and reduced charge recombination in the system [117].

Bandgap engineering through metal and nonmetal doping, along with the creation of oxygen vacancies, has proven effective in improving visible light response and overall photocatalytic efficiency. Recent advances in CaAl_2_O_4_ research have focused on developing more efficient visible‐light–active systems and improving quantum efficiency. Rare earth ion‐doped systems such as Eu‐CaAl_2_O_4_ and Dy‐CaAl_2_O_4_ introduce luminescent properties that enable photon up‐conversion and enhanced photocatalytic efficiency under visible light [118].

For instance, Ag_2_O‐modified calcium aluminate exhibited complete degradation of crystal violet (CV) within 20 min under sunlight, highlighting the strong synergistic effect of Ag_2_O in enhancing charge separation and light absorption capacity [119]. Similarly, calcium aluminate doped with europium (Eu) and neodymium (Nd), in combination with TiO_2−*x*_N_*y*_, has been investigated for nitrogen monoxide (NO) degradation. This composite system efficiently decomposed NO at a concentration of 1 ppm under a 450 W Hg lamp, showcasing the potential of rare‐earth doping in broadening the photocatalytic response to visible light [120].

Pure calcium aluminate has also been employed in the degradation of organic dyes. A study using 0.3 g/L of CaAl_2_O_4_ as a photocatalyst achieved 55% degradation of Congo red (CR) under UV light after 75 min, demonstrating moderate activity [121]. Similarly, another study reported that 5 mg of CaAl_2_O_4_ led to a 30% degradation of Methylene blue (MB) within 90 min under sunlight, indicating that its photocatalytic efficiency depends significantly on synthesis methods, crystallinity, and surface area [122].

To further enhance the photocatalytic performance, researchers have combined CaAl_2_O_4_ with other materials such as g‐C_3_N_4_ and TiO_2_. A composite of CaAl_2_O_4_: Eu^2+^, Nd^3+^/ g‐C_3_N_4_ demonstrated 56.9% degradation of Methyl orange (MO) under a 200 W Xenon lamp after 120 min, suggesting that coupling CaAl_2_O_4_ with graphitic carbon nitride improves charge transfer and extends light absorption into the visible region [123]. Furthermore, Ag‐doped CaAl_2_O_4_: Eu^2+^, Nd^3+^ combined with TiO_2_ has been studied for benzene degradation under a 75 W Hg lamp, indicating that noble metal doping and heterojunction formation significantly enhance photocatalytic activity [124].

### 3.4 Strontium Aluminate

Strontium aluminate (SrAl_2_O_4_) represents a significant member of the alkaline earth aluminate family, crystallizing in a monoclinic structure with space group P21. The crystal structure features a three‐dimensional framework of corner‐sharing AlO_4_ tetrahedra, with Sr^2+^ ions occupying the interstitial cavities [125]. This unique structural arrangement results in specific unit cell parameters: *a* = 8.447 Å, *b* = 8.816 Å, *c* = 5.163 Å, and *β* = 93.4°[126]. The compound exhibits polymorphism, with a phase transition from monoclinic to hexagonal, which significantly influences its properties and applications [127]. The material exhibits a wide bandgap of approximately 5.7 eV in its bulk form, which can be strategically modified through various approaches [128]. The valence band primarily consists of O 2p states, while the conduction band is dominated by Al 3s and 3p states [129].

The synthesis of SrAl_2_O_4_ can be accomplished through various methodologies, each offering distinct advantages in terms of product quality and characteristics. The conventional solid‐state reaction method involves high‐temperature calcination (1300°C–1500°C) of SrCO_3_ and Al_2_O_3_ precursors, resulting in well‐crystallized products but limited control over particle morphology [130]. Solution‐based methods, including sol–gel processing and co‐precipitation, operate at temperatures (900°C–1500°C) and offer superior control over particle size and morphology [131]. Novel approaches such as microwave‐assisted synthesis and solution combustion methods have emerged as energy‐efficient alternatives, providing rapid synthesis routes with enhanced control over product characteristics.

The compound also displays notable photoluminescent properties, particularly when doped with rare earth elements, making it valuable for dual‐function applications in photocatalysis and luminescence [132, 133, 134]. In terms of photocatalytic performance, SrAl_2_O_4_ demonstrates remarkable activity in various environmental applications. The material shows efficient degradation of organic pollutants, with particularly high activity under UV irradiation. The stability of SrAl_2_O_4_ in aqueous environments and its resistance to photo corrosion make it particularly suitable for water treatment applications [135]. The performance of SrAl_2_O_4_ can be significantly enhanced through various modification strategies. Doping with transition metals and rare earth elements not only enhances visible light absorption but also introduces beneficial defect states that improve photocatalytic efficiency.

**FIGURE 11:**
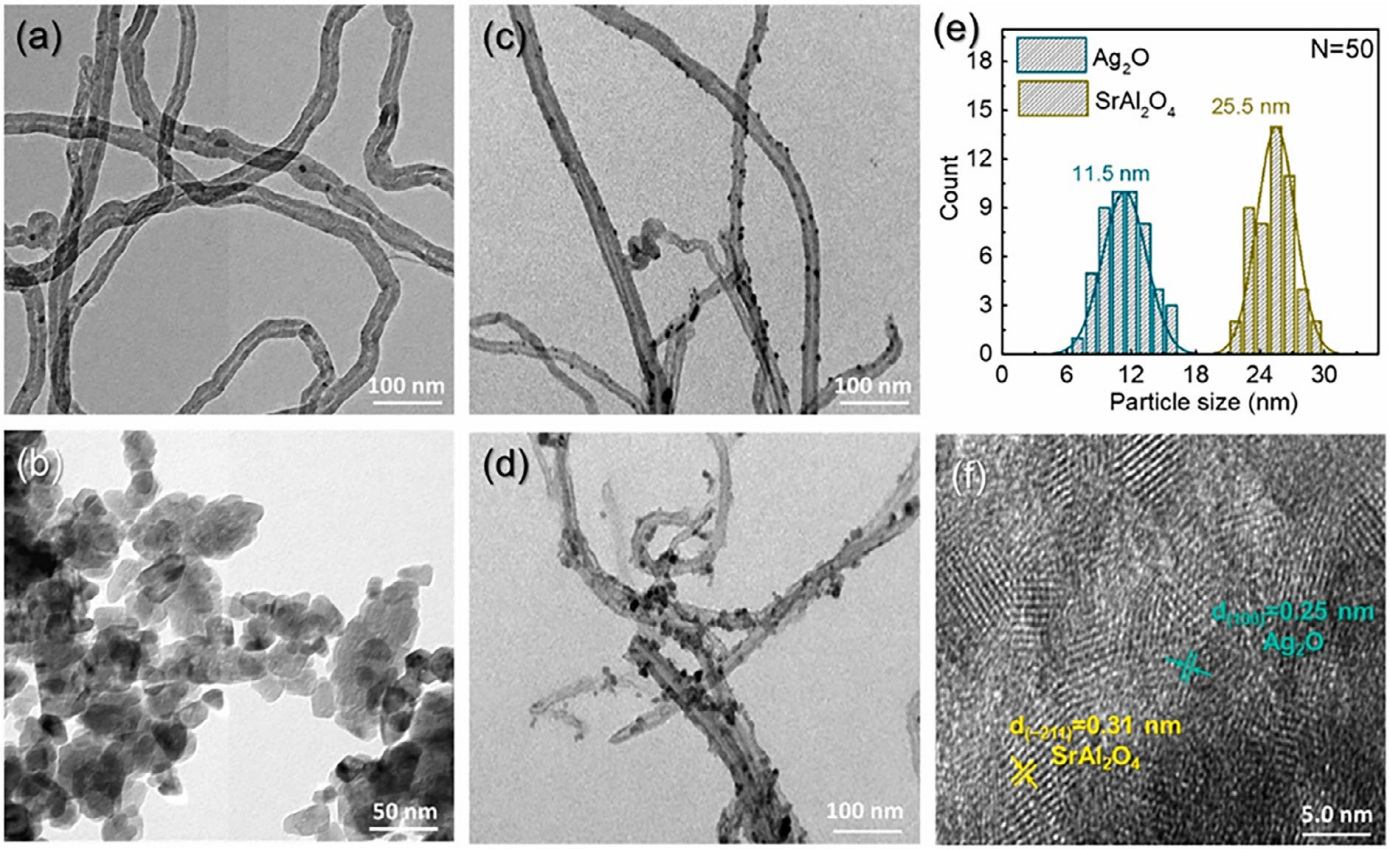
TEM images of the activated CNTs, pristine SrAl_2_O_4_, 3.0% Ag_2_O/CNT, ternary 3.0% Ag_2_O/SrAl_2_O_4_/4.0% CNT, and size histograms for Ag_2_O and SrAl_2_O_4_. High‐resolution TEM image of the selected area of 3.0% Ag_2_O/SrAl_2_O_4_/4.0% CNT (Reproduced with permission [118]. Copyright 2022, Elsevier).

The photocatalytic efficiency of various strontium aluminate‐based photocatalysts for pollutant degradation is summarized in Table 3, highlighting their performance under different experimental conditions. Mkhalid developed a visible‐light‐responsive Ag_2_O/SrAl_2_O_4_/CNT ternary photocatalyst (Figure 11) via a sol–gel method aimed at enhancing hydrogen evolution. Pure SrAl_2_O_4_, with its large bandgap (4.64 eV), was modified by doping with Ag_2_O (0.5–4 wt%) and loading carbon nanotubes (4 wt%), leading to a significant redshift in absorption edge to 481 nm and a narrowed bandgap of 2.49 eV [145]. Zhong et al. synthesized a TiO_2_/SrAl_2_O_4_:Eu^2+^, Dy^3+^ composite via the sol–gel method and demonstrated that coupling TiO_2_ with SrAl_2_O_4_:Eu^2+^, Dy^3+^ can significantly enhance its photocatalytic efficiency for the degradation of gaseous benzene. The enhancement is most notable at 1 wt% TiO_2_ loading, which exhibited 1.4 times higher photocatalytic activity compared to pure TiO_2_ [146].

**TABLE 3:** Photocatalytic performance of various strontium aluminate‐based photocatalysts for pollutant degradation.

Photocatalyst	Pollutant	Light source	Irradiation time, min	Photocatalytic degradation, %	Reference
TiO_2_/Bi‐strontium aluminate	Methylene blue	UV lamp	210	91	[136]
Bi‐SrAl_2_O_4_	Methylene blue, Congo red	UV, Sunlight	210 360	95 91	[137]
g‐C_3_N_4_–WO_3_–Bi_2_WO_6_/SrAl_2_O_4_: Eu^2+^, Dy^3+^ nanocomposite	Basic blue 41	400 W metal halide lamp	60	98	[138]
CdS‐sheathed, SrAl_2_O_4_:Eu^2+^, Dy^3+^ nanocomposites	Methyl orange	300 W Xe lamp	30	96.3	[139]
g‐C_3_N_4_/SrAl_2_O_4_: Eu, Dy/SiO_2_	Methylene blue	300 W Xe lamp	60	90	[140]
g‐C_3_N_4_@Au@SrAl_2_O_4_:Eu^2+^, Dy^3+^ Composite	Rhodamine B	300 W Xe lamp	120	80	[141]
SrAl_2_O_4_:Eu^2+^: Dy^3+^/WO_3_/polyester nanocomposite	Methylene blue	300 W lamp	90	99	[142]
SrAl_2_O_4_: x Cu	Congo red	300 W lamp	120	100	[143]
SrAl_2_O_4_: Ce: Mn	Congo red	200 W Xe lamp	300	80	[144]

García et al. developed bismuth‐doped strontium aluminate phosphors through combustion synthesis followed by post‐annealing in a carbon atmosphere and evaluated their photocatalytic potential when blended with TiO_2_. The resulting composite photocatalysts were tested for Methylene blue (MB) degradation under UV (254 nm) irradiation. Among the various doping levels studied, the TiO_2_ composite with 2.0 mol% Bi‐doped strontium aluminate exhibited the most efficient photocatalytic activity, achieving complete Methylene blue degradation within 210 min. This enhanced performance was attributed to the synergistic effect between the persistent luminescence properties of the phosphor and TiO_2_, which improved light absorption and extended photoactivation [136]. Havasi et al. investigated the photocatalytic efficiency of a ZnO: Co+ Ag nanocomposite enhanced with commercial Sr_4_Al_14_O_25_: Eu, Dy long afterglow phosphor for the degradation of methyl orange dye. The study demonstrated that incorporating this phosphor allowed the catalyst to harness and utilize persistent luminescence, enabling efficient photocatalysis under both continuous and pulsed (short on/long off) illumination conditions [147].

Mavengere and Kim synthesized a composite photocatalyst by supporting graphitic carbon nitride onto SrAl_2_O_4_: Eu, Dy using a silica binder via a colloidal‐sol coating method. The integration of g‐C_3_N_4_ onto the SrAl_2_O_4_: Eu, Dy phosphor significantly enhanced the photocatalytic performance under both germicidal UV and visible solar light. Silica improved the surface roughness and adhesion of the g‐C_3_N_4_ coating, while also modulating the photoluminescence properties by suppressing the emission peak of SrAl_2_O_4_: Eu, Dy [140]. Zargoosh and Aliabadi developed an innovative luminescent photocatalyst by integrating SrAl_2_O_4_:Eu^2+^, Dy^3+^ nanoparticles with WO_3_ and a polyester resin matrix. The SrAl_2_O_4_:Eu^2+^, Dy^3+^ particles served as persistent phosphorescent nano‐lamps, capable of emitting visible light for up to 12 h after only 5 min of exposure to 400 nm LEDs. This persistent emission effectively excited the WO_3_ photocatalyst, even in the absence of continuous external illumination. The system offered several practical advantages: low energy consumption, reusability, excellent stability, and easy recovery from solution, making it a sustainable and environmentally friendly photocatalyst [142].

## 4 Future Perspective

Despite recent progress, photocatalysts based on ZnAl_2_O_4_, MgAl_2_O_4_, CaAl_2_O_4_, and SrAl_2_O_4_ still suffer from limited visible‐light absorption and high rates of charge carrier recombination, which hinder their practical photocatalytic efficiency. To overcome these challenges, future research must emphasize innovative strategies centered on material design and modification. One promising approach involves the development of heterojunction structures particularly Z‐scheme and S‐scheme systems that promote efficient charge separation while preserving the strong redox potentials of charge carriers. Such configurations can greatly enhance photocatalytic activity under visible light. In addition, doping with transition metals or rare‐earth elements offers a viable method to tailor the electronic structure of aluminates, effectively narrowing the bandgap and extending light absorption into the visible spectrum.

Surface modification techniques, such as the introduction of oxygen vacancies or surface hydroxylation, can further facilitate the generation of reactive oxygen species necessary for pollutant degradation. Nano structuring strategies that control particle size, morphology, and surface area such as the fabrication of porous or hierarchical architectures can significantly improve photocatalyst performance by enhancing light harvesting and increasing the availability of active sites. These improvements must be supported by sustainable, scalable synthesis techniques such as sol–gel, combustion, or hydrothermal methods, which allow for precise control of physicochemical properties while maintaining environmental compatibility. Looking ahead, the integration of aluminate‐based photocatalysts into composite systems and their application in continuous flow reactors can pave the way for more efficient and practical wastewater treatment technologies. Through strategic material engineering, these systems hold great potential for advancing sustainable photocatalytic technologies.

## 5 Conclusion

Aluminate‐based materials, particularly spinel aluminates of the type MAl_2_O_4_ (where M = Zn, Mg, Ca, or Sr), have shown promising photocatalytic potential owing to their structural and electronic characteristics. Despite substantial progress achieved through controlled synthesis, doping, and composite engineering, these specific aluminates still face key limitations, most notably their wide bandgaps and the resultant restricted visible‐light absorption, along with pronounced charge‐carrier recombination, which collectively constrain their photocatalytic efficiency. Addressing these limitations requires continued research focused on optimizing material design, improving quantum efficiency, and developing scalable and eco‐friendly fabrication methods. Future advancements in this field will likely be driven by innovative approaches that integrate these materials into multifunctional systems, expanding their applicability beyond environmental remediation to energy conversion, sensing, and optoelectronics. The synergy between fundamental research and practical implementation will be crucial in unlocking the full potential of aluminate‐based photocatalysts for sustainable and efficient applications.

## Conflicts of Interest

The authors declare no conflicts of interest.
